# Complete de novo assembly of *Wolbachia* endosymbiont of *Diaphorinacitri* Kuwayama (Hemiptera: Liviidae) using long-read genome sequencing

**DOI:** 10.1038/s41598-021-03184-0

**Published:** 2022-01-07

**Authors:** Surendra Neupane, Sylvia I. Bonilla, Andrew M. Manalo, Kirsten S. Pelz-Stelinski

**Affiliations:** Entomology and Nematology Department, Citrus Research and Education Center/IFAS, University of Florida, Lake Alfred, Florida, 33850 USA

**Keywords:** Symbiosis, Genomics

## Abstract

*Wolbachia*, a gram-negative $\mathrm{\alpha }$-proteobacterium, is an endosymbiont found in some arthropods and nematodes. *Diaphorina* *citri* Kuwayama, the vector of ‘*Candidatus* Liberibacter asiaticus’ (CLas), are naturally infected with a strain of *Wolbachia* (*w*Di), which has been shown to colocalize with the bacteria pathogens CLas, the pathogen associated with huanglongbing (HLB) disease of citrus. The relationship between *w*Di and CLas is poorly understood in part because the complete genome of *w*Di has not been available. Using high-quality long-read PacBio circular consensus sequences, we present the largest complete circular *w*Di genome among supergroup-B members. The assembled circular chromosome is 1.52 megabases with 95.7% genome completeness with contamination of 1.45%, as assessed by checkM. We identified Insertion Sequences (ISs) and prophage genes scattered throughout the genomes. The proteins were annotated using Pfam, eggNOG, and COG that assigned unique domains and functions. The *w*Di genome was compared with previously sequenced *Wolbachia* genomes using pangenome and phylogenetic analyses. The availability of a complete circular chromosome of *w*Di will facilitate understanding of its role within the insect vector, which may assist in developing tools for disease management. This information also provides a baseline for understanding phylogenetic relationships among *Wolbachia* of other insect vectors.

## Introduction

The Asian citrus psyllid, *Diaphorina* *citri* Kuwayama, (Hemiptera: Liviidae), is a vector of ‘*Candidatus* Liberibacter asiaticus’ (CLas), a gram-negative $\mathrm{\alpha }$-proteobacteria that putatively causes citrus greening disease, also known as huanglongbing (HLB)^1^. *D. citri* also harbor three endosymbionts: ‘*Candidatus* Carsonella ruddii’, ‘*Candidatus* Profftella armature’, and ‘*Wolbachia*’ (*w*Di)^2^. Infected *D. citri* transmit CLas while feeding on citrus trees. Infection with CLas reduces fruit quality and yield, and eventually kills the citrus tree^1^. CLas also interacts with host *D. citri* and its endosymbionts, including *Wolbachia*, a gram-negative $\mathrm{\alpha }$-proteobacteria^3–5^. These studies reported that the abundance of *w*Di is related to the abundance of CLas in *D. citri* and regulates the phage lytic cycle genes in CLas as *D. citri* infected with “Ca. Liberibacter asiaticus” had a higher *Wolbachia* titer than the non-infected ones^5,6^. The 56-amino-acid repressor protein of *Wolbachia* in the psyllid represses SC1_gp110 (holin) gene of *Ca*. Liberibacter asiaticus which is critical for the survival of both endosymbionts in the psyllid^5^. This suggests a potential role of *Wolbachia* in CLas transmission and underscores the need for a well characterized *Wolbachia* genome^4^ in gaining a better grasp of and combating this dreadful citrus disease.

In some arthropods, such as *Drosophila* *melanogaster*, *Aedes* *aegypti*, *Culex* *pipiens*, *Acraea* *encedon*, *Armadillidium* *vulgare*, and *Asobara* *tabida*, *Wolbachia* can alter host reproduction and increase viral resistance^7–9^. The presence of *Wolbachia* can manipulate the cellular and reproductive processes by inducing cytoplasmic incompatibility, parthenogenesis, feminization, or male killing^8^. The infection of *Aedes* *aegypti* by *Wolbachia* strains, *w*MelCS (*D. melanogaster*), *w*Ri (*D. simulans*) and *w*Pip (*Culex* *quinquefasciatus*) had effects on fitness, maternal transmission, cytoplasmic incompatibility, tissue tropism and dengue virus blocking^10^. In addition, a recent study showed the importance of *Wolbachia* as *Wolbachia*-infected *A. aegypti* were resistant to Zika and dengue virus co-infection and were suitable for mitigating mosquito-borne diseases^11^. The role of *Wolbachia* in Hemiptera, including *D. citri,* remains poorly understood. The previously released draft *w*Di genome used paired-end and mate-pair Illumina datasets for the *D. citri* metagenome^12^. The draft *w*Di genome (AMZJ01000000.1) was estimated to be 1.25 Mb with 124 contigs with gaps. In this study, we utilized single molecule real-time (SMRT) sequencing by Pacific Biosciences (PacBio) technology to generate long reads from isolated *w*Di from the host cells^13^. Several challenges confronting whole genome sequencing and de novo assembly of *w*Di genome exist, including: (1) difficulties in culturing and isolating large amounts of high quality *w*Di DNA, (2) the incidence of many long repetitive elements and lateral gene transfers (LGTs) from *Wolbachia* to the host genome, and 3) the presence of Insertion Sequences (IS) and WO-prophage sequences that complicate the complete genome assembly^14–17^. The obstacles for generating a single complete contig have been overcome using long-read sequencing methods, such as PacBio, that generate longer reads through the repeats^15^. In this study, we utilized HiCanu^18^ for the complete assembly of genome sequences from *w*Di sample, which could resolve near-identical genomic repeats. The assembly resulted in a circular genome of 1.52 Mb which is the largest complete genome among assembled *Wolbachia* genomes to date among supergroup-B members, except for the complete *Wolbachia* genome from *Folsomia* *candida* (*w*Fol) of 1.8 Mb^19^ (supergroup-E), invasive cherry fruit fly *Rhagoletis* *cingulata* (*w*Cin2)^20^ of 1.53 Mb (supergroup-A). The genome dataset will enhance our ability to elucidate the interactions of *w*Di with its *D. citri* host and associated endosymbionts.

## Result and discussion

### ***w***Di genome assembly

**Figure 1:**
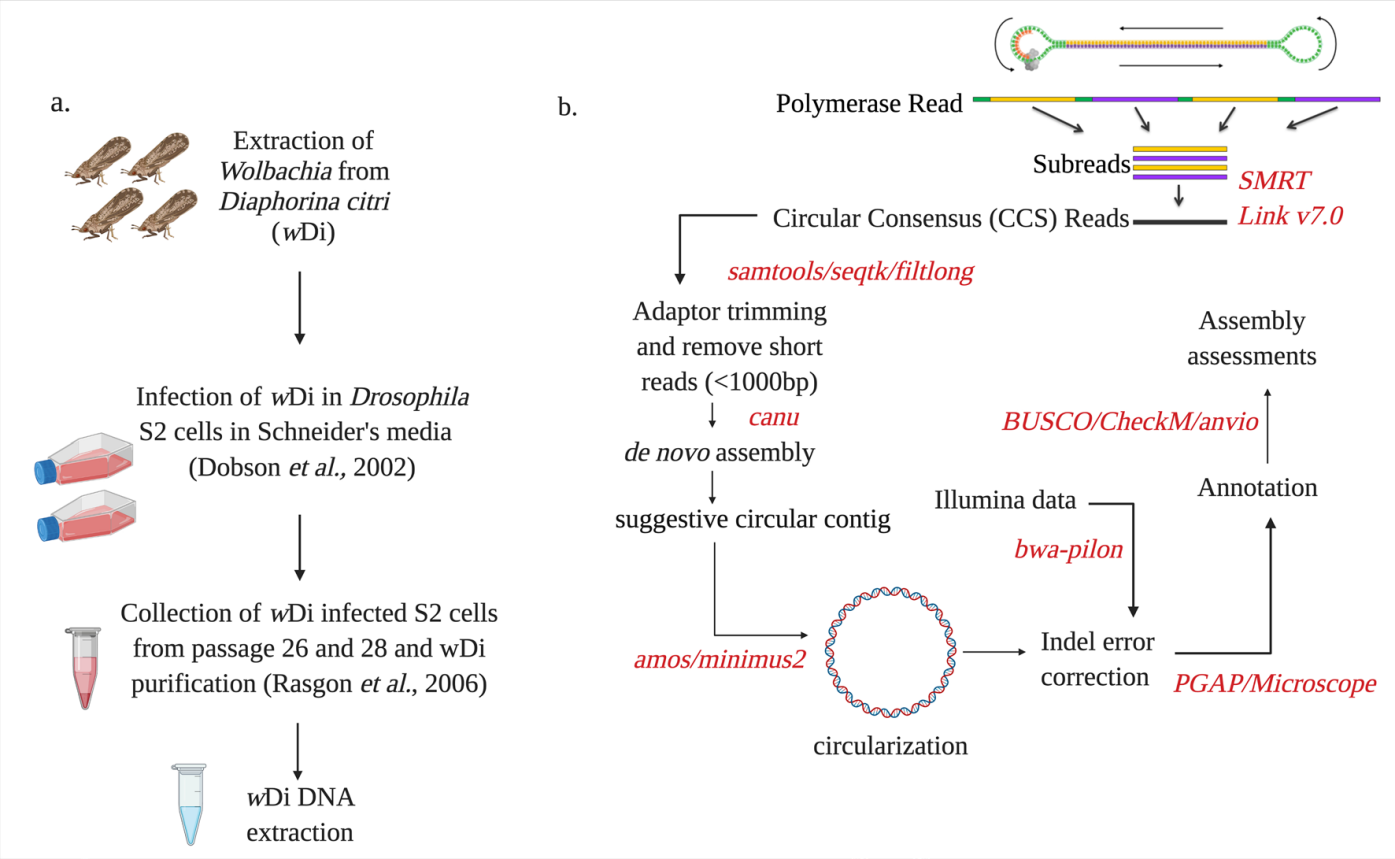
An overview of *w*Di extraction experiments and genome assembly pipeline. (**a**) A flow chart representing *w*Di extraction, culture, purification and *w*Di DNA extraction. (**b**) A flow chart showing *w*Di genome assembly pipeline.

The purpose of this study was to obtain an enclosed *Wolbachia* genome from *D. citri.* Recently, we published *w*Di genomes from a single collection point of the same *w*Di culture used in this study, which were near complete but could not be circularized^21^. The sequencing of obligate endosymbionts such as *Wolbachia* is not an easy task because of their very low abundance, inability to grow outside a host, and inability to culture axenically^22^. In addition, collection of large amounts of high-quality DNA for whole genome sequencing requires a large quantity of bacteria. This requires a high number of infected host cells to obtain the obligate endosymbiont bacteria^22^. Thus, in this study, *w*Di samples were collected from combination of two collection points (cell passages) from the same culture to obtain high quality *w*Di DNA for whole genome sequencing. An overview of *w*Di extraction and genome assembly pipeline is shown in Fig. 1.

**Figure 2:**
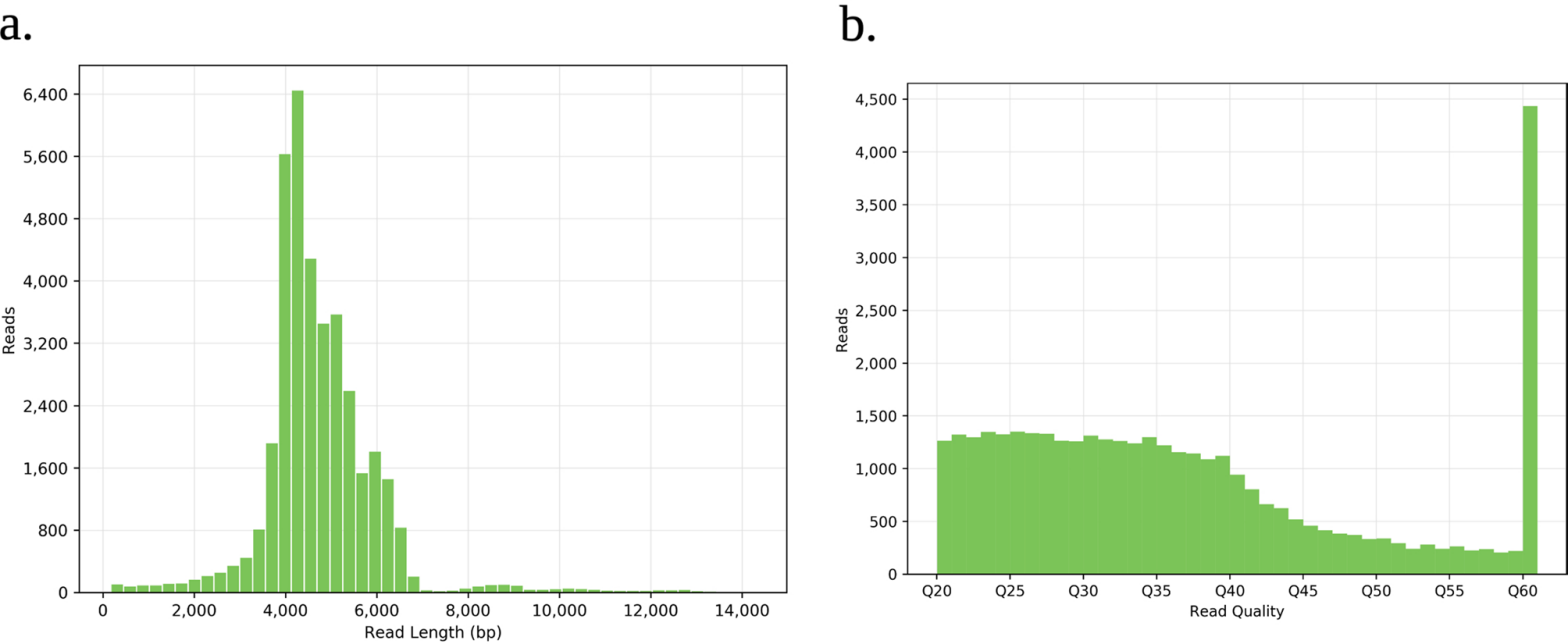
Assessment of the *w*Di genome. (**a**) and (**b**) Read length and quality assessment for PacBio circular consensus sequences for *w*Di genome.

To produce a high-quality assembly, circular consensus sequences (CCS) were used. CCS are derived accurately from the noisy individual subreads which are consensus sequence obtained from multiple passes of a single template molecule^23,24^. The raw PacBio sequencing data obtained from the SMRT cells produced 899,643 filtered subreads and a total of approximately four billion bases, with the longest subread length of 118 kb. High quality CCS reads upto 32 kb size were generated from raw PacBio reads for high quality assembly. The maximum number of CCS reads (> 4,000) generated from using SMRT® LINK v7.0 using Sequel II system were of high quality with Q60 (Fig. 2a,b). Further, 45-bp left adapter sequences were trimmed from CCS reads. In addition, the short reads < 1000 bp and worst 10% of read bases were discarded to ensure high-quality assembly with the coverage of 72.89 × . We utilized pacbio-hifi parameter in Canu v1.9 to solve the complexity of *Wolbachia* genomes and generate complete assembly with overlapping ends that can be trimmed for circularization. Pacbio-hifi, recently integrated in Canu v1.9 provides high repeat resolution than pacbio-corrected at least on complex genomes like *Wolbachia*^18^. By default, Canu v1.9 with pacbio-hifi option uses only overlaps that are below 0.03% error which is much lower than used with pacbio-corrected option. In this study, we applied an even lower rate, correctedErrorRate = 0.001, that reduces the risk for the mis-assembly. Before trimming, the assembled genome size was 1,530,940 bp. The genomes after circularization were checked for potential errors using Illumina sequencing data. Firstly, the quality of trimmed Illumina data was ensured using FastQC to determine the data quality using various quality metrics. Phred quality scores per-base for the sample was higher than 30 and GC content of 33%, following a normal distribution. The Illumina data provided median coverage of 925 × for the sample. The analyses corrected 91 SNPs, 10 small insertions totaling 73 bases, and three small deletions totaling 41 bases. The de novo assembled genome after correction was 1,528,786 bp in size with an average GC content of 34.08% (Table 1).

**Table 1:** Assembly statistics of *w*Di genome assembly.

Parameter	*w*Di
Sequencing instrument	PacBio Sequel II
Polymerase reads	72,696
Subreads	899,643
Bases (Mb)	3991.2
Mean read length (bp)	55,808
Longest subread length (bp)	118,419
CCS bases (Mb)	109.34
CCS reads (bp)	32,392
CCS coverage (×)	72.89
Assembler	Canu v1.9
Assembled chromosome (bp)	1,528,786
Circularity	Yes
G + C %	34.07
Genes	1,435
CDSs^a^ (Total)	1,394
CDSs (with protein)	1,202
Genes (RNA)	41
rRNAs	3
tRNAs	34
ncRNAs^b^	4
Pseudo genes (total)	192
PacBio accession	SRR10985324
Illumina accession	SRR11075881
GenBank accession no	CP048819
Bioproject	PRJNA603775
Project ID	SRP245886

^a^CDSs, coding DNA sequences.

^b^ncRNAs, noncoding RNAs.

The complete genome is longer than the previously reported draft contigs of *w*Di which was estimated to be 1.25 Mb^12^. The *w*Di genome is largest among assembled *Wolbachia* genomes as compared with other *Wolbachia* from arthropods and nematodes. Previously, the largest *Wolbachia* genomes were from *Folsomia* *candida* (1.8 Mb)^19^, invasive cherry fruit fly *Rhagoletis* *cingulata* (1.53 Mb)^20^ and embryos of *Aedes* *albopictus* (1.48 Mb)^25^.

### Genome annotations and assessments

**Figure 3:**
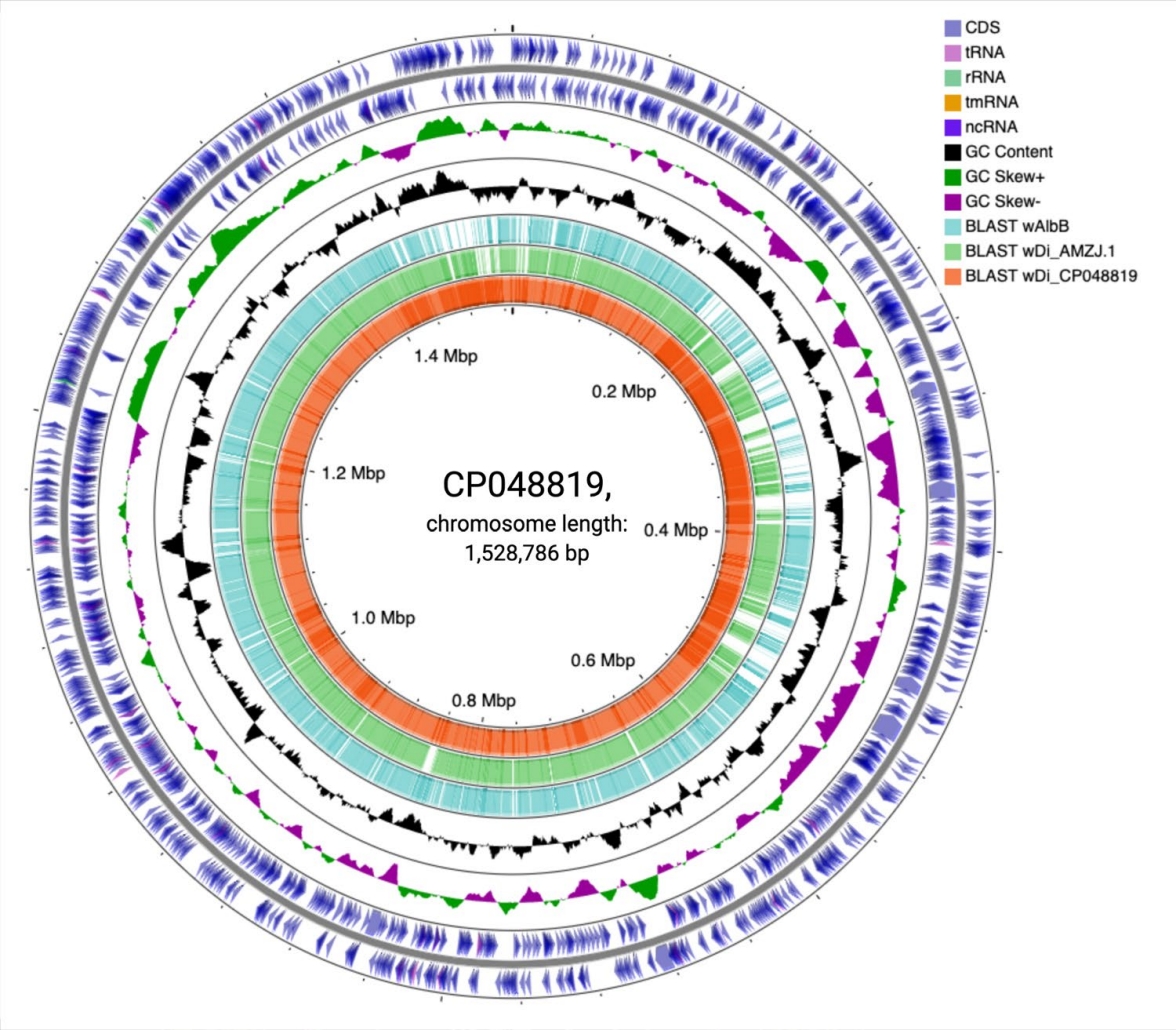
Map of the *Wolbachia* CP048819 genome prepared using CGView. Circles in order from outer to inner show following parameters: the position of coding sequences (CDS), tRNA, and rRNA genes on the positive and negative strands are denoted by circle 1 and 2, respectively. The circles 3 and 4 show plots of GC content and GC skew plotted as the deviation from the average for the entire sequence. Circles 5–7 show the positions of BLAST hits detected through BLASTn comparisons of *w*AlbB_CP031221^25^ (circle 5), *w*Di_AMZJ.1^12^ (circle 6), and itself *Wolbachia* CP048819 (circle 7).

**Figure 4:**
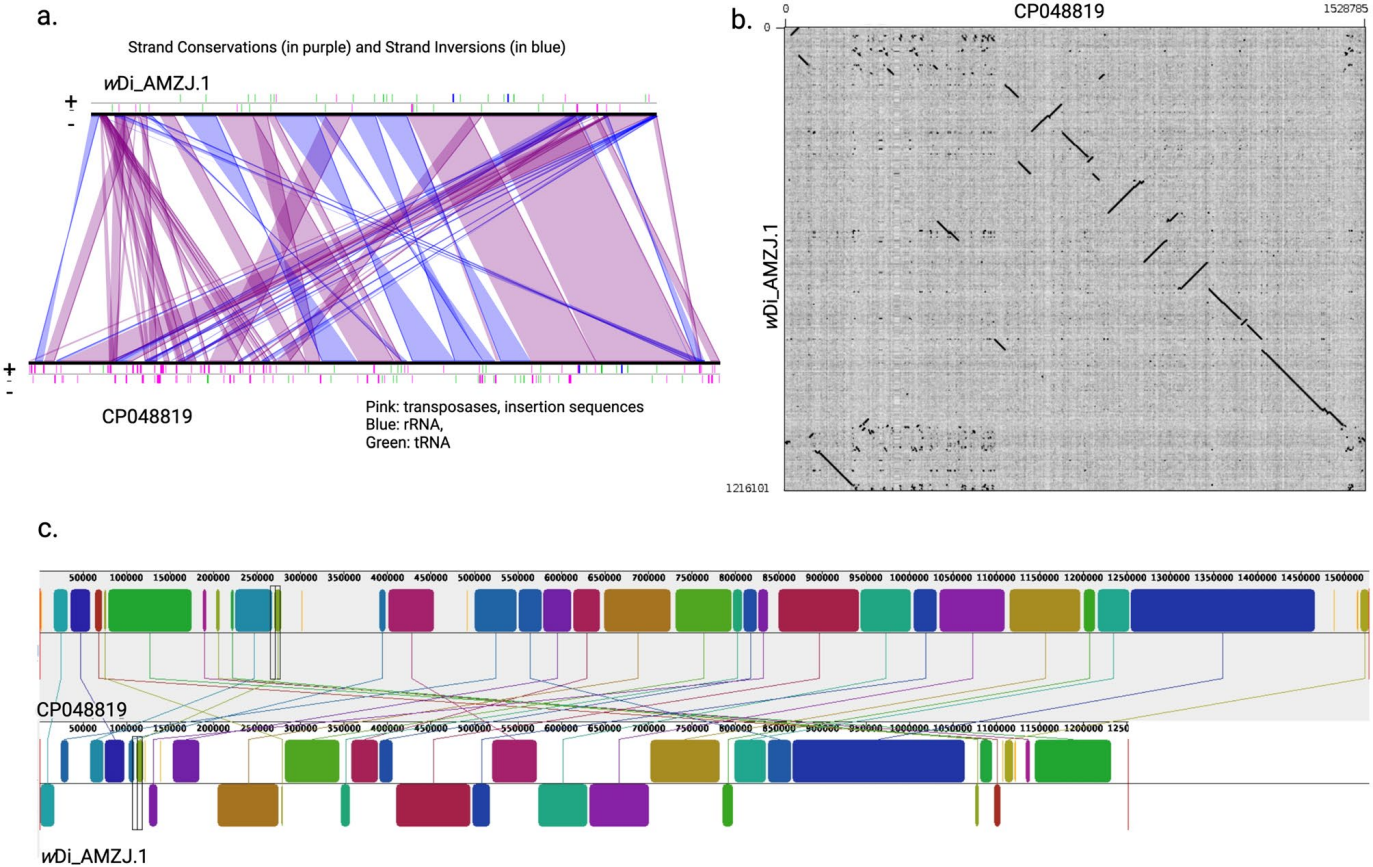
The comparison of complete *w*Di (CP048819) with draft *w*Di (*w*Di_AMZJ.1). (**a**) Lineplot (**b**) Dotplot (**c**) Mauve alignment showing thirty local colinear blocks (LCBs) on the chromosomes that were identified and joined by connecting lines in the two genomes. Few LCBs in *w*Di_AMZJ.1 are inverted, which shows reverse complement orientation.

The *w*Di genome was annotated including protein coding genes, 5S, 16S, and 23S rRNA and tRNA genes. An overview of their genome features, including CDSs, rRNAs, and tRNAs was visualized in CG view Server (Fig. 3). PGAP annotations showed assembled *w*Di chromosome to contain total of 1,435 genes which are 1,394 coding sequences with 1,202 protein coding genes. Forty-one genes are related to RNAs (three RNAs, 34 tRNAs, and four noncoding RNAs) and 192 are pseudogenes. We compared the complete *w*Di chromosome with the draft *w*Di in various perspectives using various tools implemented in Microscope platform^26^. The core genes and genome specific genes was identified comparing *w*Di_AMZJ.1^12^ based on Microscope gene families with parameter of 80% amino acid identity and 80% alignment coverage. A total of 1,073 genes were shared between two *w*Di genomes, while 239 and 183 genes were specific to *w*Di assembled in this study and *w*Di_AMZJ.1^12^, respectively, based on single transitive links (single linkage) with alignment coverage constraints and implemented in a software package (called SiLiX for SIngle LInkage Clustering of Sequences) (Figure S1; Table S1). Notably, dnaK (fragment of chaperone protein), metC (fragment of cystathionine beta-lyase/L-cysteine desulfhydrase), ylbg (putative DNA-binding transcriptional regulator), insF (transposase), rpoC (fragment of RNA polymerase subunit beta), kefB (fragment of K +: H + antiporter) constituted the largest fraction of genes in complete *w*Di. However, the Microscope platform's gene phyloprofile analysis revealed that homologs for those genes exist in draft *w*Di, with homology constraints of identity greater than or equal to 35 percent (Table S2). In complete *w*Di, tandem duplications revealed 36 locations containing 286 genes, whereas draft *w*Di revealed just 20 regions involving 64 genes. Tandem duplicated genes have an identity ≥ 35% with a minLRap ≥ 0.8 and are separated by a maximum of five consecutive genes. It is evident that tandem duplications play major role in expansion of gene families^27^. In addition, the comparison between complete and draft *w*Di was done using lineplots, dotplots, and mauve alignment. The lineplot showed the strand conservation and inversions in the syntenic regions and shows high prevalence of transposases and insertion sequences throughout the complete *w*Di genome that are absent in the draft *w*Di (Fig. 4a). The dot plot shows the breaks and inversions when compared to the draft *w*Di (Fig. 4b). Mauve alignment showed some regions in the complete *w*Di genome whose locally collinear blocks (LCBs) were absent in the draft *w*Di (Fig. 4c). Each LCB is a homologous sequence region shared by two or more of the genomes under investigation and does not contain any homologous sequence rearrangements^28^. We also looked at a number of critical elements such as transposases, Ankyrin, DNA-repair genes, and resolvases in complete and draft *w*Di that are responsible for both difficulty in assembly and genome expansion. In complete and draft *w*Di, we found 109 versus 15 transposases, 57 versus 54 proteins with ankyrin repeats, 14 versus 11 DNA repair proteins, and six versus one resolvases. The homolog for 56-amino-acid repressor protein (WP_017531870) of *Wolbachia* in the psyllid that represses SC1_gp110 (holin) gene of *Ca*. Liberibacter asiaticus was also found in the complete *w*Di genome (GZ065_v1_1041).

The BUSCO completeness scores of assembled *w*Di genome was also compared to *Wolbachia* reference genomes using bacteria_odb10 database (calculated in this study). The BUSCO completeness of the final assembled *w*Di genome showed 80.6% as compared to other reference *Wolbachia* genomes *w*Oo (78.2%), *w*Ov (78.2%), *w*Fol (81.5%), *w*AlbB (84.7%), *w*Bm (79.8%), *w*Oo (78.2%), *w*Mau (83.9%), *w*Mel (83.1%), *w*Pip (86.3%) and *w*Ri (83.9%) suggesting similar number of ‘complete and single-copy’ genes recovered in *w*Di genome compared to reference *Wolbachia* genomes and is typical and reliable for comparative genomics among *Wolbachia* genomes^25^ (Figure S2). It has been suggested that even the complete genomes of *Wolbachia* miss up to 9 to 25 genes from the BUSCO set because of their endosymbiotic lifestyle which makes genes redundant, and these genes probably are not missing from the assemblies and annotations^29^. The final assembled *w*Di genome showed 94.0% completeness when the subset database, rickettsiales_odb10 was used for the BUSCO analysis. In addition, the checkM completeness of the assembled *w*Di genome was 95.73% with 1.45% contamination. The checkM completeness and contamination falls within the range of ≥ 95% complete with ≤ 5% contamination that makes excellent reference genome for analysis^30,31^. The checkM contamination of the previously published complete *w*Fol genome (1.8 Mb)^19^ was 1.82% (calculated in this study) which was assembled from filtered reads obtained from *F. candida* genome that was sequenced using PacBio sequencing technology (Table S3). In addition, the taxonomy to *Wolbachia* sp. was confirmed using Centrifuge v1.0.3 tool that showed all sequences belonging to *Wolbachia* species.

### Insertion sequences (ISs), prophage genes, ORF7 and Ankyrin proteins

**Figure 5:**
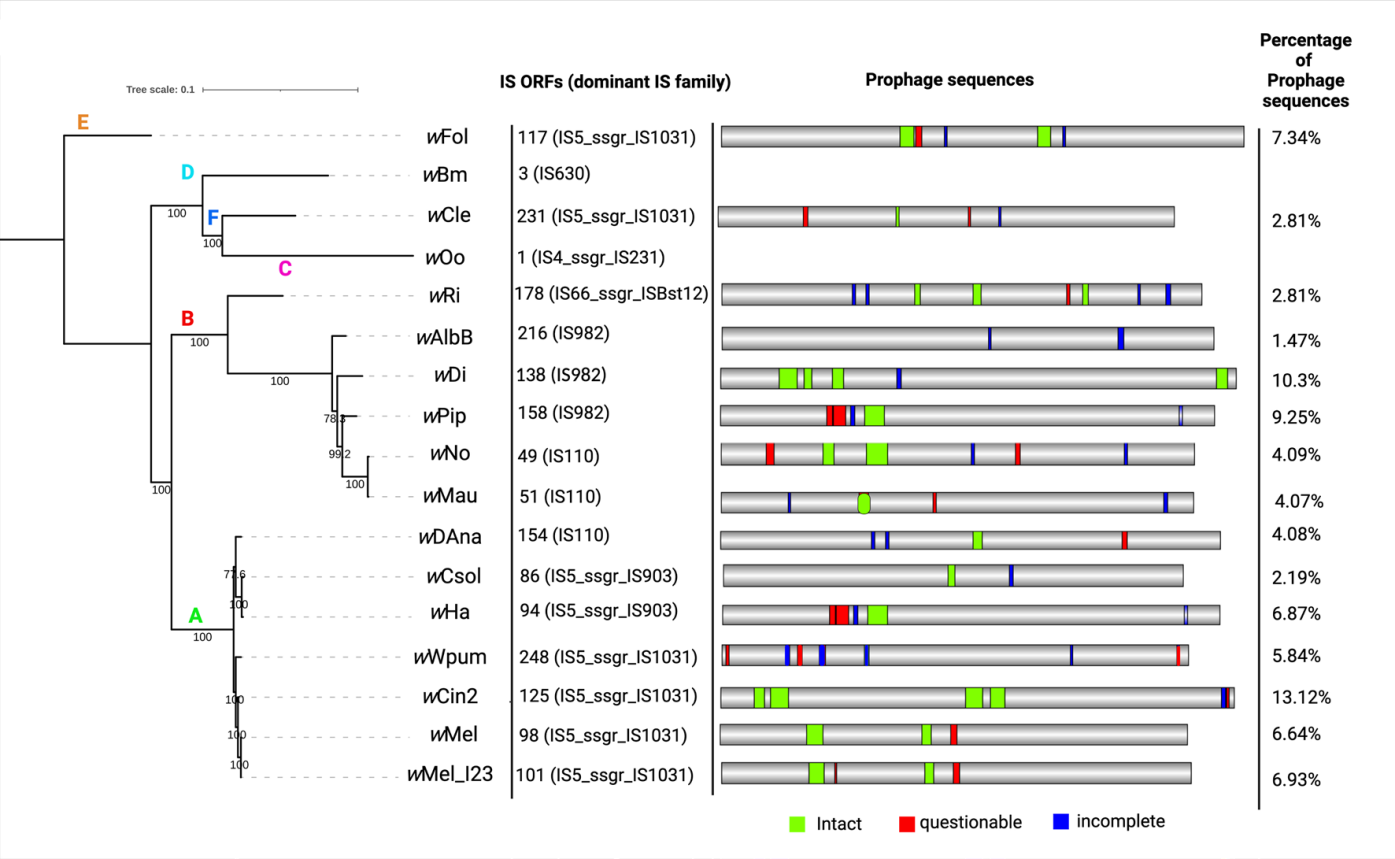
Phylogeny of complete genomes of *Wolbachia* strains belonging to supergroup A-F and schematic representation of their corresponding Insertion (IS) and prophage sequences. The maximum likelihood tree was constructed based on hmm source of single copy genes by Campbell et al.^65^ proteins using IQ-TREE v 1.6.8 and the amino acid substitution model HIVb + F + I + G4, and wFol was set as the outgroup.

Insertion sequences are bacterial class-II transposons that are capable of replication and can spread throughout the genome using cut-and-paste mechanism^32^. ISs are classified into about 20 families and play key role in genome evolution^32,33^. Specifically, 10% of the *Wolbachia* genomes consist of insertion sequence elements^34^. A total of 138 ORFs related to ISs were found in the *w*Di genome, belonging to 14 different IS families (Figure S3; Table S4). The most represented IS families were IS982 (28 copies; 20.3%), IS481 (26 copies; 18.8%), and IS110 (25 copies; 18.1%). Although the ISs in the *w*Di genome are diverse, they have less ORFs than in the entire circular *w*AlbB (CP031221) chromosome belonging to supergroup B, which has nine IS families and 216 ORFs associated to IS elements, with IS982 and IS481 having 99 and 76 copies, respectively. The other supergroup B members, *w*Pip possess IS982, *w*No and *w*Mau possessed IS110 and *w*Ri possess IS66_ssgr_ISBst12 as a dominant IS family. The majority of the members of the supergroup A, *w*Wpum, *w*Cin2, *w*Mel, *w*Mel_I23 possess IS5_ssgr_IS1031 as a dominant IS family while, *w*DAna possess IS110, *w*Csol and *w*Ha possess IS5_ssgr_IS903 as a dominant IS family. *Wolbachia* belonging to supergroups C, D that infect filarial nematodes such as *w*Oo (one IS ORF) and *w*Bm (three IS ORFs) possess highly reduced IS elements with IS4_ssgr_IS231 and IS630 as a dominant IS family, respectively. The supergroup E and F members, *w*Fol and *w*Cle possessed IS5_ssgr_IS1031 as a dominant IS family with 117 and 231 IS ORFs respectively (Fig. 5).

Prophages are subjected to selective pressure from their hosts, resulting in a variety of partial DNA genomic abnormalities such as recombination, gene loss, and progressive disintegration^35^. The prophage genes are dynamic elements that mediate horizontal gene transfer and are widespread in *Wolbachia* genomes^36,37^. Defective genomic prophages, also known as cryptic prophages, are virions that have lost their ability to generate virions and lyse host cells^35,38^. The most major difference between intact and cryptic WO is that intact WO possesses a rather complete gene module that codes for head, baseplate, and tail proteins, allowing it to generate active virions^39^.The prophage regions in the *w*Di genome showed five regions (four intact and one incomplete or cryptic) sized 55.8 kb, 23.1 kb, 32.2 kb, 11.9 kb and 34.6 kb containing 64, 33, 21, 18, and 24 proteins, respectively (Figure S2; Table S5). Altogether, prophage region constituted total of 164 prophage-associated loci scattered in four intact and one incomplete regions with the combined size of 137.9 kb (10.3%) in the *w*Di genome. Based on the existence of all genomic structures (phage attachment sites, genes encoding structural phage proteins, and genes coding for proteins involved in DNA regulation, insertion to the host genome, and lysis), the four entire WO*w*Di phages have the ability to create virions. One cryptic WO*w*Di sized 11.8 kb (location: 522,438–534,303) lacks phage baseplate and tail assembly proteins. *w*Di genome supports widely held belief that *Wolbachia* with cryptic prophages usually has at least one intact WO prophage^40^. This shows the expansion of the prophage region when compared to other supergroup B members such as *w*AlbB_CP031221 (1.47%), *w*No (4.09%), *w*Mau (4.07%) and but comparable to *w*Pip (1.48 Mb genome size) with 9.25% prophage sequences (with only one 59.8 kb sized intact prophage region with other four cryptic prophage regions) (Fig. 5). Surprisingly, PHASTER analysis revealed two cryptic prophage regions of 6.4 kb and 15.4 kb in *w*AlbB_CP031221 without the presence of intact prophage region. However, four WO-like islands (designated *w*AlbB WO like island 01 through *w*AlbB WO like island 04) and 19 prophage-associated loci (13 CDS, 6 pseudogenes) were discovered by BLAST comparisons to several WO phages totaling 111 prophage-associated loci with a combined size of 116 kb (8%) without active prophages^25^. Other *Wolbachia* genome only with cryptic prophages were found in group A member, *w*Wpum (*Wolbachia* in *Wiebesia* *pumilae*)^39^ having no ability to produce active virions.

The WO prophage areas are sometimes used in cytoplasmic incompatibility genetic investigations^41^. The BLASTp searches of WOMelB WD0631 (NCBI accession number AAS14330.1) and WD0632 (AAS14331.1) in Microscope platform for *CifA* and *CifB* protein sequences, respectively^41^ found no homologs in the *w*Di strain for *CifA* but a few for *CifB*. Among *CifB* hits using HHpred^42^, GZ065_v1_1517, GZ065_v1_0240 follow Module B-1 (ModB-1 with PDDEXK nuclease family, and various other restriction endonucleases such as NucS, HSDR_N, and MmeI), and GZ065_v1_0695, GZ065_v1_0696, GZ065_v1_0704 follow Module B-3 [with ubiquitin-modification (Ulp-1) and protease-like domains (Sentrin-specific protease)]^41^.

In addition, the *w*Di genome revealed the presence of four different minor capsid gene ORF7 paralogs (GZ065_00870, GZ065_01245, GZ065_01575, and GZ065_6965) (Figure S3) as in *Nasonia* *vitripennis* A *Wolbachia*^37^ which are present in the four different prophage sequence regions. The protein domain annotations of the assembled genomes showed 57 (4.0%) proteins in the *w*Di genome to contain at least one copy of an ankyrin repeat domain (Figures S3; Table S6) which is comparable to ANK proteins *w*Mel, *w*Ri, and *w*Pip with about 4% of the total genes^43^. These ANK proteins of about 33 amino acids play significant role in interactions between host and symbionts^34,44^ and are found abundantly in genes of WO-prophage^44^.

Many contemporary hypotheses propose that obligate endosymbionts should have limited genome sizes^45^, similar to *Wolbachia* strains in filarial nematodes, which contain no or few insertion sequences, transposable elements, and prophage sequences, due to their obligate association with the host^46^. Recent study have shown that the genome of the obligatory *w*Fol^29^ strain, on the other hand, is the biggest complete *Wolbachia* genome ever identified, with 1,801,626 base pairs (bp) and highly enriched in repeated and mobile elements (124 transposases, 96 ankyrin repeat proteins, 34 DNA-repair genes, and 19 resolvases). In *w*Di too, the genome is highly enriched in repeated and mobile elements (109 transposases, 57 proteins with ankyrin repeats, 14 DNA repair proteins, and six resolvases) than other supergroup-B members^29^. All known *Wolbachia* strains are in a similar transitional stage, in which they are primarily vertically transferred and do not exist in specialized structures^47^. As a result, their genome size is expected to vary depending on the host^47^.

### COG, eggNOG, and pfam annotations

COG automatic classification revealed 1,092 CDSs classified in at least one COG group in the *w*Di genome (Table S7). eggNOG annotations of protein coding genes assigned functions to 1,221 protein coding genes (Table S8). The top five pathways were related to “replication, recombination and repair”, “translation, ribosomal structure and biogenesis”, “energy production and conversion”, “posttranslational modification, protein turnover, chaperones”, and “coenzyme transport and metabolism”. The Pathway Tools was used to observe whether the metabolic pathways were complete or not. The analysis showed 40 complete metabolic pathways and 62 incomplete metabolic pathways (Table S9). The pfam annotation of *w*Di identified 1075 protein coding genes with unique pfam domains. The important pfam domains for mobile genetic elements such as DDE Transposase domain DDE_Tnp_1 (PF01609), DDE_Tnp_1_3 (PF13612), DDE_Tnp_4 (PF13359), DDE_Tnp_IS240 (PF13610.6), Retroviral Integrase domain rve (PF00665), rve_3 (PF13683), and reverse transcriptase domain RVT_1 (PF00078) were found abundantly in *w*Di genome (Table S10).

### Toxin-antitoxin system and Type IV Secretion SSystem (T4SS) genes

Toxin–antitoxin (TA) systems are genetic components that consist of a toxin gene (proteins) and its antitoxin counterpart (protein or non-coding RNAs). In bacteria various processes, like translation, replication, cytoskeleton development, membrane integrity, and cell wall biosynthesis are affected by TA toxins^48^. PGAP annotation in the *w*Di genome revealed the presence of Type II RelE/ParE toxin genes, GZ065_00055, GZ065_03670 (pseudogene) and one Type II RatA family toxin gene, GZ065_04425. Based on the BLASTp search using *w*Pip antitoxin gene, WP_007302904.1, we identified GZ065_00050 as a possible antitoxin gene for RelE toxin. Type II RatA family toxin gene, GZ065_04425 was situated immediate to ssrS noncoding RNA gene (Rfam RF00013), separated by fewer than 18 nucleotides. Previously, RelE/ParE and RatA/ssrS toxin-antioxin modules were also reported in *w*Cle, *w*Fol, *w*Pip, *w*Mel, *w*Ri, *w*Au, *w*Ha, *w*No^49^.

Genes related to the Type IV Secretion System (T4SS) are another important group represented in *Wolbachia*. Bacteria utilize T4SSs to proliferate and survive inside the host secreting protein effectors, protein-DNA complexes^50^. The *w*Di genome revealed the presence of 14 genes associated to T4SSs (Table S11). These genes were organized in two operons in each *w*Di genome. Operon 1 contains *virB8*, *virB9-1*, *virB10*, *virB11*, and *virD4*. Operon 2 contains *virB3*, *virB4*, *virB6-1*, and *virB6-2*. The *virB2* and *virB7* genes were found to be scattered elsewhere in the genomes. Interestingly, we found both *virB2* (three copies) and *virB7* (one copy) genes in the *w*Di genome. These genes have been reported as absent among *Wolbachia* and most members of the order *Rickettsiales*^51,52^. However, recent studies have shown the presence of *virB2* gene (pilus component) in *Wolbachia* *pipientis* from *Ae. albopictus* (*w*AlbB)^25^, *Wolbachia* from *Laodelphax* *striatellus*^53^*, Candidatus* *Wolbachia* *bourtzisii* (*w*DacA), *Wolbachia* *pipientis* *w*DacB from *Dactylopius* *coccus*^54^, and *Wolbachia* from *Muscidifurax* *uniraptor* (*w*Uni)^55^. In addition, the *virB7* gene (pilus-associated protein) was previously observed only in *Wolbachia* from *Laodelphax* *striatellus* (*w*Stri)^53^. Bing et al.^53^ also showed *w*Di clustered together with *w*Stri with a strong support in a monophyletic clade and suggested that these strains shared the same ancestor.

### Comparative genomics of wDi with reference *Wolbachia* genomes

**Figure 6:**
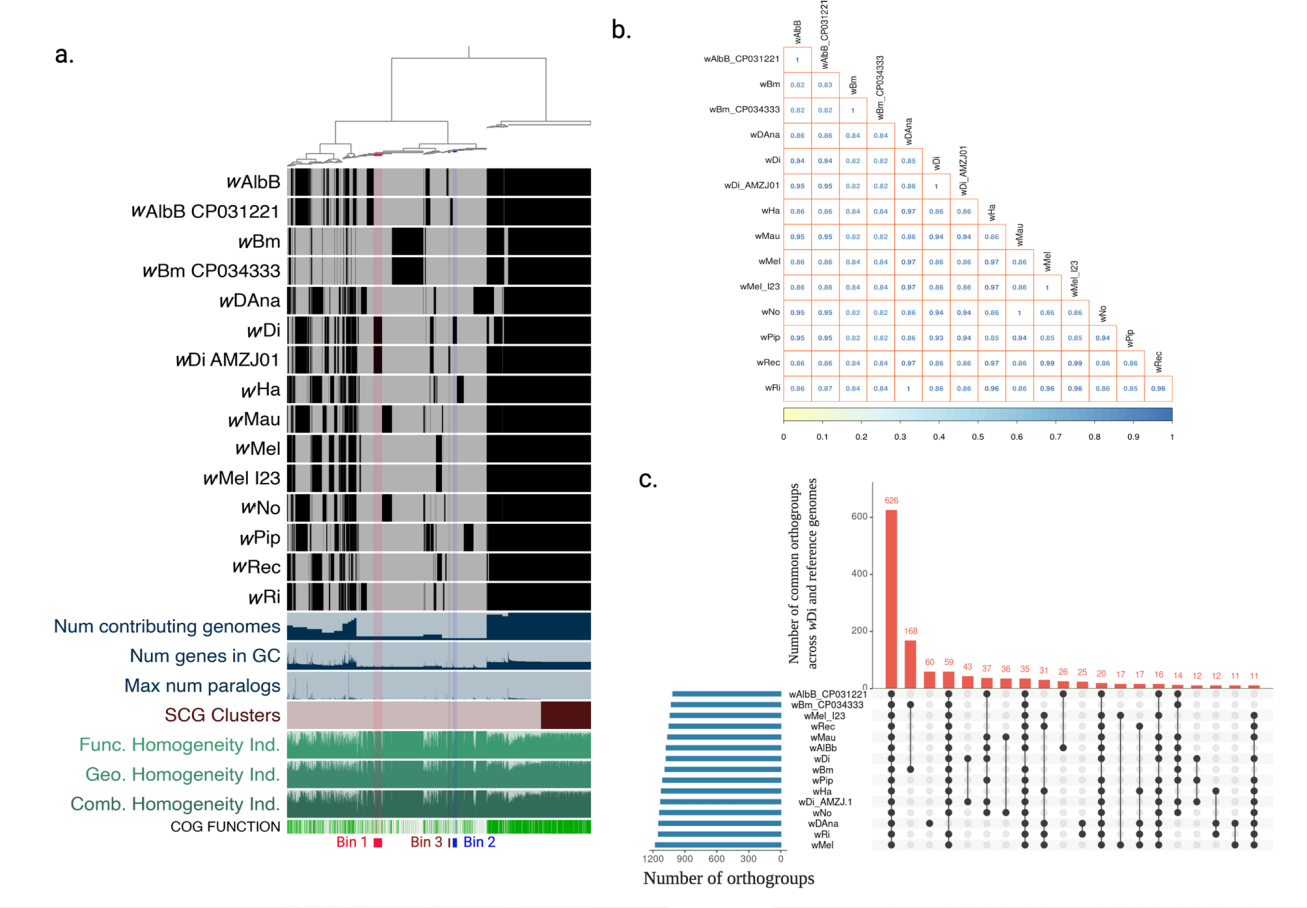
Comparative genomics of *Wolbachia* genomes. (**a**) *Wolbachia* metapangenome representing 2,112 gene clusters with 18,800 genes that were identified in 15 *Wolbachia* genomes. The metapangenome represent following parameters: combined homogeneity index, geometric homogeneity index, functional homogeneity index, Single-copy Core Genes (SCG) clusters, maximum number of paralogs, number of genes in gene cluster (GC), number of genome gene clusters that have hits. Regions of the map shown in black denote similar content between genomes. The dendrograms on the top represents the hierarchical clustering of genomes based on the occurrence of gene clusters. (**b**) The Average Nucleotide Identity (ANI) between the *w*Di genome and 14 genomes of *Wolbachia* evaluated using the ‘anvi-compute-ani’ which utilizes PyANI^56^ in ‘ANIb’ mode to compute average nucleotide identity across the genomes anvio v5.5.0^57^. (**c**) UpSet plot showing number of common orthogroups across *w*Di and reference *Wolbachia* genomes. 626 orthogroups were present in all *Wolbachia* strains analyzed which is represented by the first bar. The fifth bar represents 43 orthogroups unique to *w*Di genomes. The black and gray dots represent the presence and absence of orthogroups, respectively, in each *Wolbachia*.

The *Wolbachia* pangenome describes 2,112 gene clusters with 18,800 genes that were identified in 15 *Wolbachia* genomes. The pangenome study resulted three bins that were unique to *w*Di genomes. The Bin_1 consisted of 58 gene clusters with 127 genes common in both complete and incomplete *w*Di_AMZJ.1^12^ genomes, Bin_2 consisted of 29 gene clusters with 62 genes that were unique to the complete *w*Di genome, and Bin_3 consisted of 12 gene clusters with 13 genes that were unique to incomplete *w*Di_AMZJ.1^12^ genome (Fig. 6a, Table S12). The largest fraction of genes in three bins constituted Ankyrin repeat proteins (n = 28; play important role in interactions between host and symbionts) and IS4 transposase (n = 11; play role in DNA mobility using “cut and paste” mechanism), chromosome segregation ATPases (n = 5; play important role in chromosome condensation and segregation during cytoplasmic incompatibility in male insects), curved DNA-binding protein CbpA, containing a DnaJ-like domain (n = 2; act as a molecular chaperone in an adaptive response to environmental stresses other than heat shock), DNA repair protein RadC (n = 2), DNA-directed RNA polymerase (n = 2), RecA-family ATPase (n = 6) , REP element-mobilizing transposase (n = 2), transcriptional regulator with XRE-family HTH domain (n = 2), Mg/Co/Ni transporter MgtE (n = 2; important in inorganic ion transport and metabolism) and rest were conserved protein with unknown function.

The ANI values among the *w*Di genome and reference *Wolbachia* genomes indicated the similarity in the range of 82% (supergroup D-*w*Bm) to 95% (supergroup B-*w*AlbB) and 99.8% to incomplete *w*Di_AMZJ.1^12^ genome (Fig. 6b). OrthoFinder assigned 21,264 genes (96.3% of total) to 1,924 orthogroups (Table S13) in the 15 *Wolbachia* genomes. There were 626 orthogroups with all species present and 407 of these consisted entirely of single-copy genes (Fig. 6c). The analysis showed 43 orthogroups unique to complete and draft *w*Di genomes.

### Phylogenetics of *w*Di and other *Wolbachia* genomes

**Figure 7:**
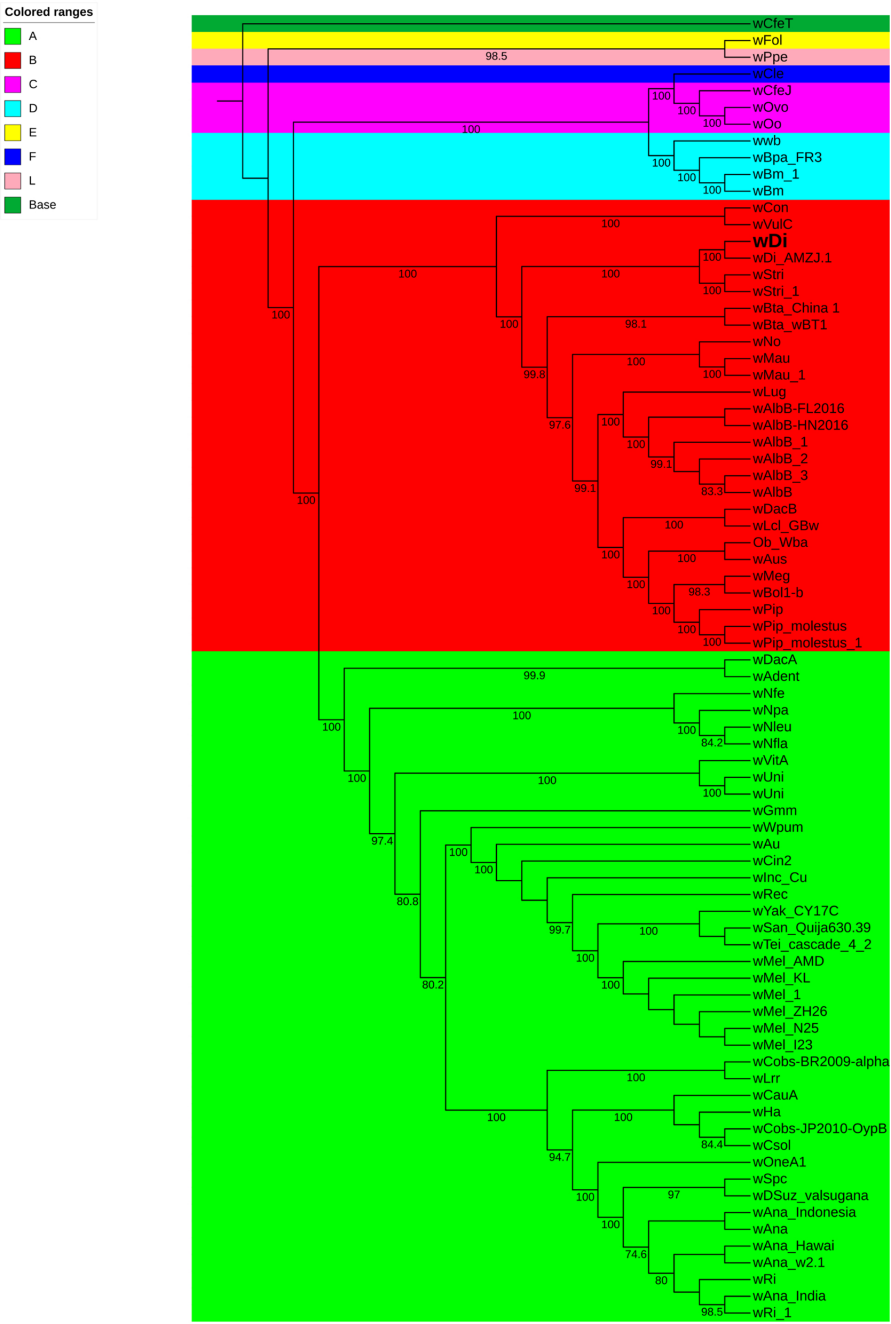
Phylogenetic relationship of *Wolbachia* genomes using concatenated protein sequences of single copy genes obtained from each genome using hmm source of single copy genes by Campbell et al.^65^. The total of 78 Wolbachia genomes including wDi genome sequenced in this study was used. The maximum likelihood tree was constructed using IQ-TREE v 1.6.8^66^ using ultrafast bootsrap mode with 5000 iterations. Branch support was estimated using the Shimodaira–Hasegawa (SH)-like approximate likelihood ratio test with 1,000 replicates. The amino acid substitution model HIVb + F + I + G4 was used and wFol was set as the outgroup. The bootstrap values > 50% are shown at the respective node. The Wolbachia supergroups are color coded which are shown in color ranges.

The IQ-TREE v 1.6.8 tool was used to construct a ML phylogenetic tree using the concatenated protein sequences of single copy genes including ribosomal proteins of reference *Wolbachia* genomes obtained from NCBI database (Table S14) with the *w*Di genome. The single copy genes were utilized instead of multilocus sequence typing loci (*gatB*, *coxA*, *hcpA*, *fbpA*, and *ftsZ*)^58^ which are problematic in phylogenetic analyses and may not accurately represent the properties of different *Wolbachia* strains^59^. The advent of sequencing technology and availability of complete and draft genomes of *Wolbachia*, recent phylogenetic studies have been done utilizing single copy gene sets^53,59,60^ rather than whole-genome sequence typing^61^. Although comparisons of whole *Wolbachia* genome sequences is useful for strain differentiation, diversity estimates, and phylogenetic analyses, the size is cumbersome and not necessary to answer specific questions that can be addressed using genetic marker loci^59^. The obtained tree (Fig. 7) indicated that the *w*Di genome belonged to supergroup-B *Wolbachia* strains (*w*VulC, *w*Con, *w*Lug, *w*Bta, *w*Stri, *w*AlbB, *w*DacB, *w*Lcl, *w*No, *w*Mau, *w*Aus, Ob_Wba, *w*Bol1-b, *w*Meg, and *w*Pip) and made a clade with *w*Stri (the *Wolbachia* from Korean *Laodelphax* *striatellus* population) and *w*Stri_1 (the *Wolbachia* from Chinese *L. striatellus* population). *Wolbachia* are supergrouped (A, B, E–H), the *Wolbachia* endosymbionts of arthropods belong to supergroup-A and -B and of filarial nematodes belong to supergroup-C and -D^8,62^. *w*Ppe belongs to supergroup-L^63^, whereas *w*CfeT strain is ancestrally to most other *Wolbachia* lineages (used as an outgroup)^64^. The phylogenetic analysis by Saha et al.^12^ also indicated that *Wolbachia* from *D. citri* belongs to supergroup-B using *FtsZ* and *Wsp* genes.

## Conclusions

The genome sequence of the *Wolbachia* culture isolated from *D. citri* was completely assembled and compared with other *Wolbachia* genomes available in the NCBI database. This study is in accordance with the study by Sinha et al.^25^, which demonstrated that high quality, complete *Wolbachia* genome assemblies can be achieved from long-read sequences of high coverage without enrichment, such as through Large Enriched Fragment Targeted Sequencing^67^ and other target genome enrichment techniques^68,69^. In this study, we used DNA from an axenic *Wolbachia* cultures for whole genome sequencing rather than filtering *Wolbachia* sequence reads from the whole insect genome sequence. The latter, referred to as a metagenomic sequencing approach, is a frequent practice that generates low coverage reads for *Wolbachia* genome assembly^70,71^. Recent integration of the pacbio-hifi option in Canu (HiCanu) facilitates generation of complete assemblies consisting of repeat resolution on complex genomes like that of *Wolbachia* rather than pacbio-corrected assemblies in previous versions. In addition, concatenated protein sequences of single copy genes generated using hmm source from Campbell et al*.*^65^ delineated supergroup-B *Wolbachia* of *D. citri* from other supergroups. The availability of a complete circular genome of the *D. citri* endosymbiont, *Wolbachia,* will facilitate the development of endosymbiont-mediated strategies for pest and disease management. This study expands the list of complete *Wolbachia* reference genomes that can be useful in studying evolutionary relationships among *Wolbachia* of arthropods and nematodes.

## Materials and methods

### Extraction of *Wolbachia* from *D. citri* (*w*Di)

*D. citri* were collected from a laboratory culture established in 2005 from a population collected in Polk Co. (28.0′ N, 81.9′ W), Lake Alfred, Florida, USA. Individual psyllids were placed on sterile diet rings for two days prior to *Wolbachia* extraction. The surface sterilized psyllid was homogenized in 1.0 mL of Schneider’s *Drosophila* (S2) medium (catalog number 21720024, Gibco) followed by centrifugation at 100 × g for five minutes. The supernatant was further centrifuged at 400 × g for five minutes to pellet *w*Di with insect debris. The pellet was resuspended with 1.0 mL of S2 medium separate *w*Di from impurities. The samples were centrifuged at 100 × g for five minutes to pellet impurities, and the supernatant was transferred to a new tube. The final centrifuge step was conducted at 4000 × g for five minutes, and the pelleted *w*Di was resuspended in fresh 1.0 mL of S2 media.

### Infection of *w*Di in S2 cells and isolation of *w*Di from cell culture

*Drosophila* S2 cells (catalog number R69007, Invitrogen) were infected with *Wolbachia* extracted from *Diaphorina* *citri* (S2 + *w*Di)^72^ and maintained in Schneider’s *Drosophila* medium (catalog number 21720024, Gibco) containing 10% heat inactivated fetal bovine serum (catalog number 10082147, Gibco); 50 units of penicillin and 50 μg streptomycin sulfate (catalog number 15070063, Gibco) per mL (S2 complete media) Dobson et al*.*^73^ according to standard procedures^74^. The S2 + *w*Di cells were harvested and lysed by vortex using 3 mm borosilicate glass beads to isolate *w*Di. The supernatant samples were processed as described by Rasgon et al*.*^75^. *w*Di cells from the same culture were collected on different dates (different cell passages, 26 and 28) and combined to obtain enough *w*Di DNA to produce a complete genome^21^.

### ***w***Di Genomic DNA (gDNA) extraction

The *w*Di gDNA was extracted using the MagAttract HMW DNA Mini kit (catalog number 67563, Qiagen) using manufacturer’s protocol with few modifications. The modifications were as follows: The bacterial pellet was resuspended in 180 µl ATL buffer [from DNeasy® Blood and Tissue Kit (catalog number 69506, Qiagen)] with 20 µl Proteinase K and incubated for 30 min at 56 °C. 15 μl MagAttract Suspension and 280 μl Buffer MB was added to the sample and mixed by pulse vortexing. The sample tubes were transferred to the tube holder of the Magnetic Rack (without the magnetic insert). The tube holder of the Magnetic Rack (without the magnetic insert) was placed onto the mixer and incubate at room temperature (15–25 °C) for 3 min at 1400 rpm. The magnetic insert was placed into the tube holder of the Magnetic Rack, wait (~ 1 min) until bead separation has been completed, and the supernatant was removed. The extracted gDNA was purified using the DNeasy PowerClean Cleanup kit (catalog number 1287750, Qiagen). gDNA was quantified using the Qubit 1 × dsDNA HS Assay kit (ThermoFisher Scientific) and DNA quality was assessed using the TapeStation Genomic DNA ScreenTape (Agilent Technologies).

### Long-read (PacBio) sequencing

Sequencing of *w*Di gDNA was performed on six replicate samples (five samples are not included in this study). *w*Di gDNA (4–8 µg in 150 µl TE) was sheared down to 10 kb using Covaris g-TUBES (catalog number 520079, Covaris Inc.), using two passes at 7,000 rpm. The resulting size of the fragments was verified on the TapeStation Genomic DNA ScreenTape (Agilent Technologies). Barcoded, 10 kb insert-size libraries were constructed using 600–700 ng of pure and fragmented (10 kb) from each bacterial sample using the protocol of PacBio for multiplex SMRT sequencing of bacterial genomes (PacBio Manual PN 101–069-200–02) in conjunction with barcodes from the Barcoded Adaptor Kit 8A (PacBio PN 101–081-300). Briefly, the library construction reactions consisted of the following sequential steps: ExoVII treatment, DNA Damage Repair, End Repair and Blunt-end ligation of barcoded SMRT bell adaptors. After ligation, samples were pooled, purified using AMPure, and treated with ExoIII/ExoVII to eliminate excess adaptors and any damaged DNA. This procedure resulted in ~ 800 ng of adaptor ligated SMRT bell library. The final library was further size selected in the SageELF™ instrument (catalog number ELD7510), using 0.75% agarose gel cassettes and the 1–18 kb v2 cassette definition program. The desired SageELF™ fractions in the 5–20 kb range, averaging 10 kb (TapeStation) were cleaned using AMPure magnetic beads (0.6:1.0 beads to sample ratio) and eluted in 15 μl of 10 nM Tris HCl, pH 8.0. The library size selection by ELF step yielded 126 ng of ready-to-sequence material. Sequencing was performed on the PacBio SEQUEL instrument using the Chemistry 3.0 reagents in combination with the SMRT® LINK v 6.0 software. The library was added on the PacBio SEQUEL sample plate at 8 pM by diffusion-loading and 224 min pre-extension time for sequencing in LR-SMRT cells with 20-h data collection. All other steps for sequencing were done according to the recommended protocol by PacBio sequencing calculator.

### Short-read (Illumina) sequencing

The gDNA samples for Illumina sequencing were fragmented using the Covaris to 400 bp following the manufacturer recommended protocol. The genomic libraries were constructed using 100 ng as the input and the NEBNext Ultra II DNA library prep kit for Illumina (New England Biolabs). Three PCR cycles were performed with each library prior to library validation using the TapeStation High Sensitivity D5000 ScreenTape (Agilent Technologies). Libraries were quantified using the Qubit 1 × dsDNA HS Assay kit (ThermoFisher Scientific) and molar concentration was calculated to pool the libraries in equimolar ratios. The pool was then quantified and 14 pM was loaded into the MiSeq flow cell. The run was set as a 300 paired-end run using the 600-cycles v3 kit.

###  De novo genome assembly

PacBio CCS were generated using SMRT® LINK v7.0 using Sequel II system. The parameters used for CCS generation were minimum full passes of three and minimum predicted accuracy of 99%. The left adapter sequences (45 bp) were trimmed using seqtk (https://github.com/lh3/seqtk). The reads smaller than 1000 bp were filtered out using filtlong (–min_length 1000, –keep_percent 90) (https://github.com/rrwick/Filtlong). The de novo assembly was done using Canu v1.9 (https://github.com/marbl/canu)^76^ using the “pacbio-hifi” option^18^. The suggested circular chromosome was rendered using the following parameters: trim-assemble, genomeSize = 1.5 m, correctedErrorRate = 0.001, cnsErrorRate = 0.050, minReadLength = 3000. The resulted contig was circularized by introducing a ‘break’ in the single contig using Amos v3.1.0 and Minimus2 (http://amos.sourceforge.net/wiki/index.php/Minimus2) that trimmed the duplicate sequences in the beginning and end of the chromosome to produce a circular genome. The origin of replication was adjusted using Circlator v1.5.5^77^.

### Genome correction

The PacBio-only assembled genome can have a high probability of indel errors^78^. Therefore, the assembled genome was checked for potential errors using Illumina data obtained from respective samples using the Pilon error-detection and correction tool^79^. The adapters and low-quality Illumina sequences were filtered using program Trimmomatic v0.36 (ILLUMINACLIP: adapters.fasta:2:30:20 LEADING:3 TRAILING:3 SLIDINGWINDOW:4:15 MINLEN:50)^80^. The quality of trimmed reads was assessed using FastQC v0.11.7^81^. After cleaning, the reads were mapped to the PacBio chromosome using bwa v0.7.17^82^ using pair-end mode. The indexed bam output file obtained from bwa was utilized for indel correction using Pilon v1.22^79^.

### Genome annotations and assessments

Genome annotation was done using the standard NCBI Prokaryotic Genome Annotation Pipeline (PGAP)^83^ and Microscope platform^26^. PGAP annotations are available at NCBI GenBank. The annotations from Microscope platform were used for some comparative studies and mentioned when discussed below (represented by GZ065_v1_n). The completeness of the genome was assessed using Benchmarking Universal Single-Copy Orthologs (BUSCO) v4 using bacteria_obd10 database (Creation date: 2019–06-26, number of species: 4085, number of BUSCOs: 124) and rickettsiales_odb10 database (Creation date: 2020–03-06, number of species: 34, number of BUSCOs: 364)^84^ and CheckM^85^. Microscope platform was utilized for completeness using CheckM, Clusters of Orthologous Groups (COG) classification of proteins including functional annotation of protein-coding genes using eggNOG-Mapper v1.0.3^86^ , eggNOG database v4.5.1^87^, encoded pathway analysis via Pathway Tools v23^88^ and the MicroCyc metabolic pathways database^89^. The map of the circular genome with gene feature information was generated using CGView^90^. The SiLiX software^91^ integrated in the Microscope platform that uses the MicroScope gene families (MICFAM) was used for the analysis of the components (core-genome, strain specific sequences) for complete and draft wDi. MAUVE^28^ was used for complete and draft *w*Di genomes alignments with locally collinear blocks. Gepard^92^ was used for creating dot plot between complete and draft *w*Di genomes. LinePlot tool implemented in the Microscope platform was used to create a line plot for a global comparison, based on minimum synton size of eight genes. Protein sequences from Microscope platform were used for identifying Pfam domains using pfam_scan.pl script v1.5 (last accessed March 10, 2020) using Pfam database v31.0^93^. The prophage regions were identified by PHAge Search Tool Enhanced Release (PHASTER. https://phaster.ca/)^94^ (last accessed September 28, 2021). ISsaga web server http://issaga.biotoul.fr/issaga_index.php^95^(last accessed September 28, 2021) was used to find Insertion Sequence (IS) elements using ISfinder database^33^. HHpred^42^ was used for the detection of protein domains for identification of modules^41^ to categorize the possible cytoplasmic incompatibility genes. ORF7, or phage WO-B genome was identified from Pfam which are molecular markers for *Wolbachia* strain typing^96,97^ and plays a possible role in inducing cytoplasmic incompatibility^98^. The prophage sequences, IS elements, Ankyrin genes, T4SS genes and ORF7 sequences in the corresponding *w*Di genomes was represented in a circos plot using Circa (OMGenomics, http://omgenomics.com/circa/).

### Comparative genomics of wDi genome with other *Wolbachia* genomes

#### *Wolbachia* metapangenome, ANI identity, and orthogroup analyses

The assembled *w*Di genome from this study was compared to various reference genomes: *w*Pip^99^, *w*AlbB^100^, *w*AlbB_CP031221^25^, *w*Mel^44^, *w*Bm_CP034333^67^, *w*Bm^101^, *w*Mau^67^, *w*Ri^34^, *w*DAna^102^, *w*Ha^22^, *w*Mel_I23^70^, *w*No^22^ and *w*Rec^103^. The previously published, non-circular *w*Di genomes *w*Di_AMZJ.1^12^ was also included in the comparison. The pangenome analyses were performed using anvio v5.5.0^57^ (http://merenlab.org/software/anvio/). The taxonomy was assigned using Centrifuge v1.0.3^104^. The COGs to the reference genomes were assigned using program ‘anvi-run-ncbi-cogs’. The program ‘anvi-pan-genome’ was used following flags and parameters: ‘-use-ncbi-blast’, ‘-minbit 0.5’, and ‘-mcl-inflation 5’ for the *w*Di genome and reference genomes. The similarity between the *w*Di and reference genomes were calculated using ‘anvi-compute-ani’ which utilizes PyANI^56^ in ‘ANIb’ mode to compute average nucleotide identity across the genomes. The orthogroups across the *w*Di and reference genomes were identified using Orthofinder v2.4.0^105^ and common orthogroups across multiple genomes were visualized via UpSet plot using Intervene (https://asntech.shinyapps.io/intervene/)^106^.

### Phylogenetic analysis

We constructed two maximum likelihood phylogenetic trees in different scale. The phylogenetic analysis was performed using protein sequences hits obtained via ‘anvi-get-sequences-for-hmm-hits,’ which utilizes the hidden markov model (hmm) source from Campbell et al.^65^ using 139 single copy genes including 48 ribosomal genes. One small scale phylogenetic tree was constructed using seventeen complete *Wolbachia* chromosomes for studying and visualizing the abundance and variations of Insertion and prophage sequences. For big scale phylogenetic tree, seventy-seven *Wolbachia* genomes (taxid: 953) were downloaded from the NCBI database using command ncbi-genome-download to perform the phylogenetic analysis with *w*Di genome. The concatenated protein sequences of single copy genes were aligned using MUSCLE^107^ and were subjected to ModelFinder^108^ for RAxML tree using Bayesian Information Criterion (BIC). The best amino acid substitution model was used for construction of maximum likelihood phylogenetic tree using IQ-TREE v1.6.8^66^ using ultrafast bootstrap mode with 5000 iterations. Branch support was estimated using the Shimodaira–Hasegawa (SH)-like approximate likelihood ratio test with 1,000 replicates. Modelfinder and IQ-TREE was integrated in a PhyloSuite v1.2.2 software^109^ The rerooting, labeling, and color coding of the phylogenetic tree was performed using iTOL v5.7 (https://itol.embl.de/)^110^.

## Supplementary Information

Supplementary Information 1.

Supplementary Information 2.

Supplementary Information 3.

Supplementary Information 4.

Supplementary Information 5.

Supplementary Information 6.

Supplementary Information 7.

Supplementary Information 8.

Supplementary Information 9.

Supplementary Information 10.

Supplementary Information 11.

Supplementary Information 12.

Supplementary Information 13.

Supplementary Information 14.

## Data Availability

The accessions SRR10985324, and SRR11075881 under Bioproject PRJNA603775 connected with biosample SAMN13940805 have been deposited at the NCBI. The assembled genome and annotations have been deposited at the NCBI GenBank database under the accession CP048819. All the supplemental materials have been uploaded in Figshare: 10.6084/m9.figshare.14397131. Figure S1. Venn diagram showing common and genome specific genes between complete *w*Di and draft *w*Di_AMZJ.1 genome. Figure S2. BUSCO assessment of the completeness of *w*Di genomes with reference sequences. Figure S3. Circos plot representation of various features in the *w*Di genome. The *w*Di genome is represented by the outer circle. The first, second, third, fourth and fifth inner circle represents the track for IS elements, Ankyrin genes, T4SS genes, prophage sequences, and ORF7 sequences, respectively in the *w*Di genome. Table S1 shows list of complete and draft *w*Di genome specific genes. Table S2 shows list of orthologs of complete and draft *w*Di using annotation from Microscope platform. Table S3 shows list of *Wolbachia* genomes sequenced and assembled using different technology and assembly tools. Table S4 shows Insertion Sequences (ISs) in the *w*Di genome. Table S5 shows prophage statistics in the *w*Di genome. Table S6 shows list of Ankyrin genes in the *w*Di genome. Table S7 shows COG automatic classification of protein coding genes in the *w*Di genome. Table S8 shows eggNOG annotations of protein coding genes in the *w*Di genome. Table S9 shows Metabolic pathways analysis in the *w*Di genome. Table S10 shows Pfam domain annotations for the *w*Di proteins of the *w*Di genome. Table S11 shows list of genes related to Type IV Secretion System in the *w*Di genome. Table S12 shows summary of *Wolbachia* Pan gene clusters. Table S13 shows Orthogroup analyses. Table S14 shows list of *Wolbachia* genome assemblies downloaded from the NCBI database, consisting of 139 single copy genes including 48 ribosomal genes from Campbell et al.^65^ used for the hidden markov model (hmm) source, concatenated protein sequences, and phylogenetic tree construction file.
